# Age-Associated Differences in Ductal Involvement in Biliary Lithiasis: A Consecutive MRCP Cohort Study

**DOI:** 10.3390/life16081361

**Published:** 2026-08-19

**Authors:** Lucian Mihai Florescu, Mădălin Mămuleanu, Ioana Andreea Cîrlig, Dumitru Rădulescu, Alesandra Florescu, Mihai-Alexandru Ene, Aurelia Ștefania Domenco, Alexandru Marian Olaru, Alexandra Gabriela Cosmina Țâru, Raluca Elena Nica, Rossy Vlăduț Teică, Ștefan Paitici, Ioana Andreea Gheonea

**Affiliations:** 1 Department of Radiology and Medical Imaging, University of Medicine and Pharmacy of Craiova, 200349 Craiova, Romania; lucian.florescu@umfcv.ro (L.M.F.); raluca.nica@umfcv.ro (R.E.N.); rossy.teica@umfcv.ro (R.V.T.); ioana.gheonea@umfcv.ro (I.A.G.); 2 Department of Automatic Control and Electronics, University of Craiova, 200585 Craiova, Romania; madalin.mamuleanu@edu.ucv.ro; 3 Doctoral School, University of Medicine and Pharmacy of Craiova, 200349 Craiova, Romania; andreea.cirlig97@gmail.com (I.A.C.); mihai.ene@umfcv.ro (M.-A.E.); domenco.aura@gmail.com (A.Ș.D.); 4 Department of Surgery, University of Medicine and Pharmacy of Craiova, 200349 Craiova, Romania; stefanppaitici@gmail.com; 5 Department of Rheumatology, University of Medicine and Pharmacy of Craiova, 200349 Craiova, Romania; alesandra.florescu@umfcv.ro; 6 Department of Radiology and Medical Imaging, Emergency Clinical County Hospital of Craiova, 200642 Craiova, Romania; alexandra.taru@yahoo.com

**Keywords:** MRCP, choledocholithiasis, gallstones, bile duct diameter, aging, phenotype shift, logistic regression

## Abstract

**Background**: Magnetic resonance cholangiopancreatography (MRCP) enables non-invasive assessment of biliary lithiasis, duct caliber, and associated anatomical findings. Although age-dependent reference limits for common bile duct (CBD) diameter have been described, the relationships among age, gallbladder stone morphology, ductal anatomy, and CBD stones remain incompletely characterized in consecutive radiology cohorts. This study assessed age-associated MRCP patterns and imaging features associated with CBD stones. **Methods**: We retrospectively analyzed 242 consecutive MRCP examinations performed for biliary indications. Recorded variables included age, sex, gallbladder and CBD stone characteristics, CBD diameter, intrahepatic bile duct (IHBD) dilatation, CBD tortuosity, cystic duct morphology, periampullary diverticulum, microlithiasis, and associated pancreatobiliary findings. Ordered trend tests, group comparisons, correlation analyses, ROC analysis, and a parsimonious multivariable logistic model were used. Model 2 included six predictor parameters for 65 CBD-stone events (10.8 events per variable) and was internally validated with 2000 bootstrap resamples. **Results**: The cohort had a median age of 63 years (IQR 49–73), and 135 patients (55.8%) were female. GB stones were present in 211 patients (87.2%), and CBD stones in 65 (26.9%). Across ordered age strata, GB stone prevalence decreased (*p* = 0.006), whereas CBD stone prevalence increased (*p* = 0.038); CBD diameter and IHBD severity also increased with age (both *p* < 0.001). CBD-positive patients had larger CBD diameters than CBD-negative patients (median 10.0 vs. 6.5 mm, *p* < 0.001). In Model 2, CBD diameter was the strongest independent imaging correlate of CBD stones (OR 1.567 per 1 mm, 95% CI 1.310–1.873; *p* < 0.001), whereas age was not independently associated after adjustment (*p* = 0.781). CBD diameter alone achieved an AUC of 0.853; Model 2 achieved an AUC of 0.896 and an optimism-corrected AUC of 0.884. **Conclusions**: Older age groups showed greater ductal involvement and larger CBD caliber in this consecutive MRCP cohort. CBD diameter was the strongest imaging correlate of CBD stones, while age and CBD diameter provided overlapping information in the adjusted model. These findings describe an observational MRCP pattern and require external validation.

## 1. Introduction

Gallstone disease is among the most common gastrointestinal disorders and becomes increasingly prevalent with age [1]. Although most gallstones remain asymptomatic, biliary lithiasis may lead to cholecystitis, cholangitis, pancreatitis, biliary obstruction, gallstone ileus, or gallbladder perforation, thereby imposing a substantial clinical and healthcare burden [1,2]. Established risk factors include age, female sex, obesity, family history, estrogen exposure, dietary factors, and rapid weight loss [2]. Most gallstones are cholesterol-predominant, and their formation reflects interactions among bile supersaturation, gallbladder hypomotility, nucleation factors, genetic susceptibility, and environmental influences [2,3,4,5,6,7,8,9,10,11].

Ultrasonography is the first-line imaging test for gallbladder stones, but evaluation of the distal CBD may be limited. CT is frequently used in acute care, yet small or isoattenuating CBD stones may be missed [1,2,6]. MRCP provides non-invasive, high-resolution assessment of the biliary tree and can characterize stone burden, duct caliber, and anatomical variants relevant to management [7].

CBD caliber is influenced by age. Ultrasound and MRCP studies in asymptomatic populations have shown gradual age-related widening, and recent population-based MRCP data have proposed age-dependent upper reference limits [8,9,10]. These reference studies help distinguish physiological variation from obstruction, but they do not establish how age-related caliber differences relate to gallbladder stone morphology, CBD stones, and additional ductal anatomy in patients referred for biliary indications.

Few consecutive radiology cohorts have evaluated these variables together. We therefore aimed to describe age-associated MRCP patterns of biliary lithiasis and identify imaging features independently associated with CBD stones. CBD stone status was defined by consensus MRCP interpretation; systematic ERCP, EUS, or surgical confirmation was not available. Accordingly, the study was designed to assess imaging associations rather than diagnostic accuracy or causal pathways.

## 2. Materials and Methods

### 2.1. Ethical Approval

This retrospective study was conducted in accordance with the Declaration of Helsinki and was approved by the Committee of Ethics and Academic and Scientific Deontology of the University of Medicine and Pharmacy of Craiova, Romania (approval no. 163/03.07.2026).

### 2.2. Study Design and Population

Consecutive MRCP examinations performed for biliary indications between February 2023 and May 2026 (n = 312) were retrospectively screened. Inclusion criteria were: (1) MRCP performed for a biliary indication; (2) diagnostic image quality allowing reliable assessment of CBD caliber and biliary lithiasis; and (3) availability of core demographic and imaging variables, including sex, age, CBD diameter, and stone-related variables.

**Figure 1:**
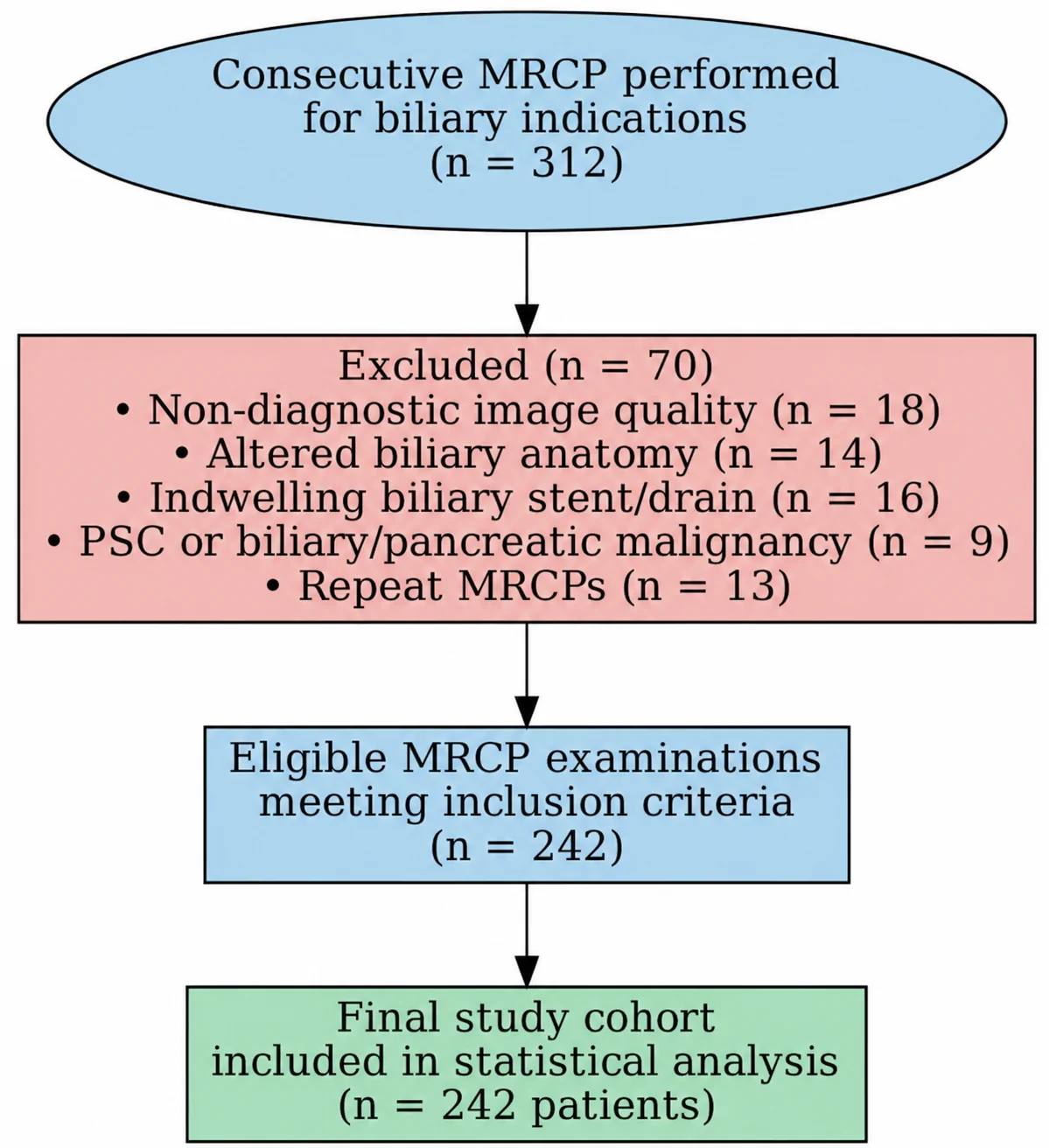
Flowchart of patient selection and eligibility criteria.

Examinations were excluded if they were non-diagnostic due to severe motion artifacts or incomplete biliary coverage (n = 18), demonstrated altered biliary anatomy precluding standard duct assessment, such as biliary-enteric anastomosis (n = 14), showed an indwelling biliary stent or drain at the time of MRCP (n = 16), or had known primary sclerosing cholangitis or biliary/pancreatic malignancy as the dominant cause of obstruction (n = 9). Repeat MRCP examinations in the same patient were also excluded, retaining only the first eligible examination (n = 13). After application of these criteria, 242 patients formed the final study cohort (Figure 1).

### 2.3. MRI Protocol

All examinations were performed on a 1.5-T magnetic resonance imaging system (GE Healthcare, Milwaukee, WI, USA) using a phased-array body coil.

The MRCP protocol included standard localization sequences followed by multiplanar anatomical and cholangiographic acquisitions. The imaging protocol consisted of coronal T2-weighted fat-suppressed images, axial T2-weighted images, axial T2-weighted fat-suppressed images, axial T1-weighted images, diffusion-weighted imaging (DWI) with corresponding apparent diffusion coefficient (ADC) maps, and a respiratory-triggered three-dimensional (3D) MRCP sequence acquired in the coronal plane.

For patients who received intravenous gadolinium-based contrast administration, additional sequences included native axial T1-weighted fat-suppressed images followed by dynamic post-contrast axial T1-weighted fat-suppressed acquisitions obtained during the arterial, portal venous, and delayed phases.

MRCP images and source datasets were reconstructed and reviewed using the institutional Picture Archiving and Communication System (PACS). Evaluation focused on biliary duct morphology, common bile duct caliber, intrahepatic bile duct dilatation, stone burden and location, cystic duct anatomy, gallbladder morphology, and associated pancreatobiliary findings.

All MRCP examinations were independently reviewed by two senior radiologists with dedicated experience in hepatobiliary imaging. Disagreements were resolved by consensus during a joint review session, and the consensus MRCP assessment served as the imaging reference for this analysis. ERCP, EUS, surgery, and clinical follow-up were available only for subsets of patients and were not used as a uniform reference standard. Imaging variables were extracted according to predefined morphological criteria and standardized review principles. Cases with non-diagnostic image quality were excluded during eligibility assessment.

### 2.4. Image Review and Imaging Variables

Recorded variables were divided into several categories:

#### 2.4.1. Demographic Variables Included Age and Sex

Those variables were included because both are among the most consistently reported demographic determinants of gallstone disease. Age was analyzed both as a continuous variable and further categorized into the following ordered strata: <20, 20–39, 40–59, 60–79, and ≥80 years.

#### 2.4.2. Biliary Duct Morphology

**Figure 2:**
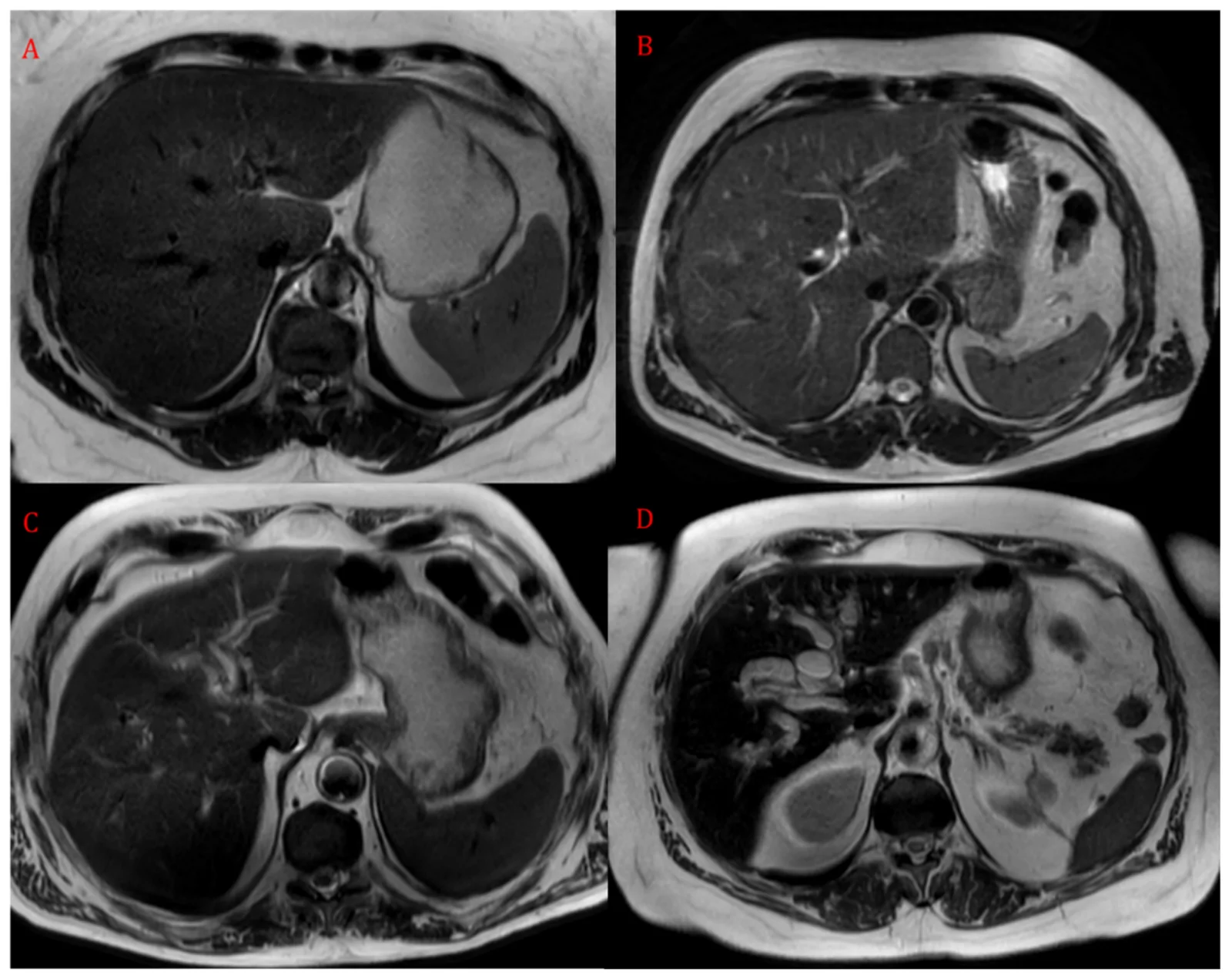
Axial T2-weighted MR images illustrating IHBD dilatation: (**A**) absent, grade 0; (**B**) mild, grade 1; (**C**) moderate, grade 2; and (**D**) severe, grade 3.

Biliary duct morphology was assessed by measuring CBD diameter (mm) and by evaluating intrahepatic bile duct (IHBD) dilatation and CBD tortuosity; (IHBD) dilatation was assessed visually and graded using a four-point scale (0 = absent, 1 = mild, 2 = moderate, and 3 = severe) as illustrated in Figure 2.

**Figure 3:**
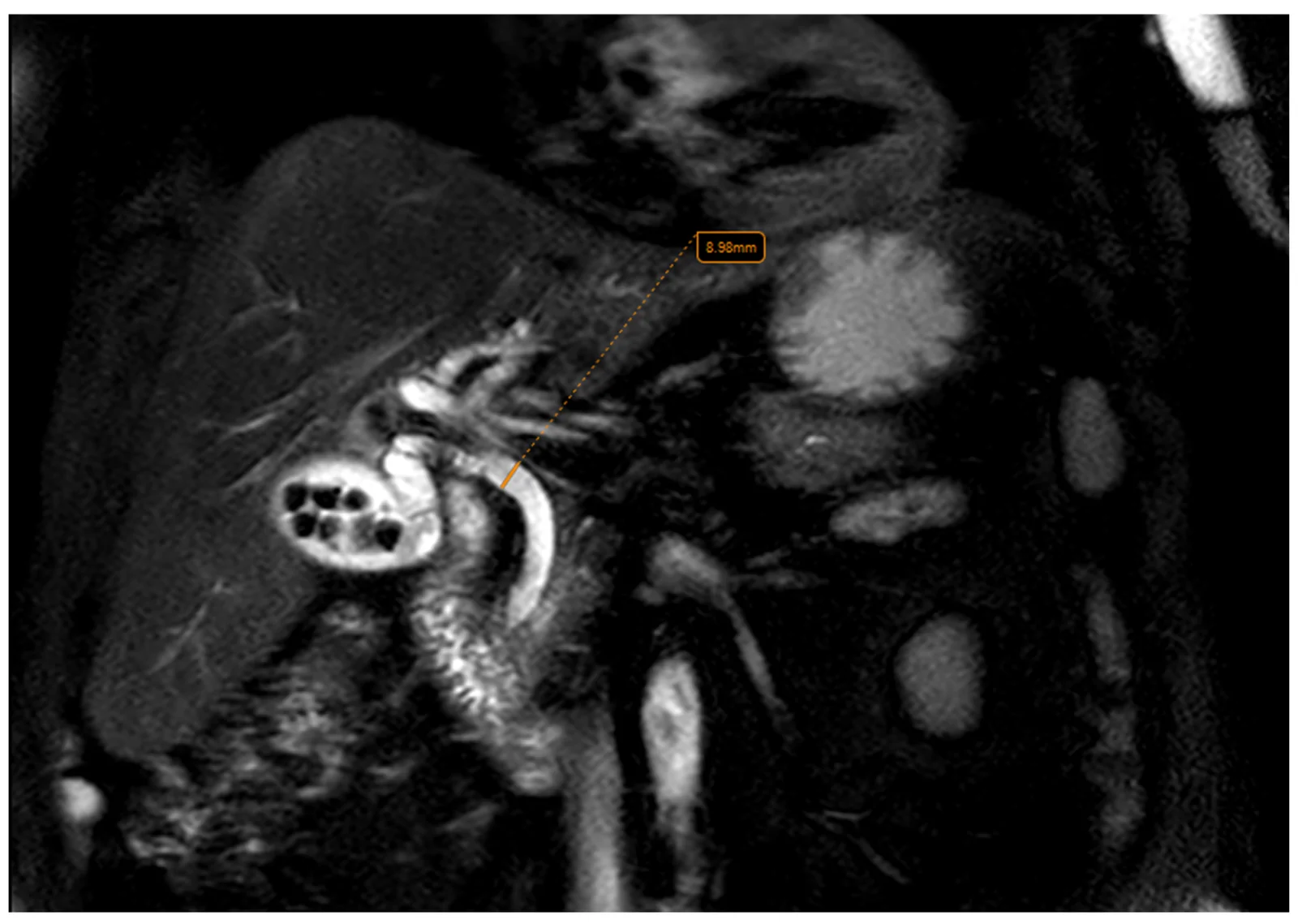
Coronal T2-weighted image illustrating CBD diameter measurement.

CBD diameter was measured in millimeters at the proximal level, immediately below the confluence between the cystic duct and the common hepatic duct, as shown in Figure 3.

CBD tortuosity was evaluated using the PACS angle-measurement tool and classified on a three-point scale as follows: 0, rectilinear or mildly C-shaped course; 1, one distinct angulation of ≤120°; and 2, at least two distinct angulations of ≤120°.

**Figure 4:**
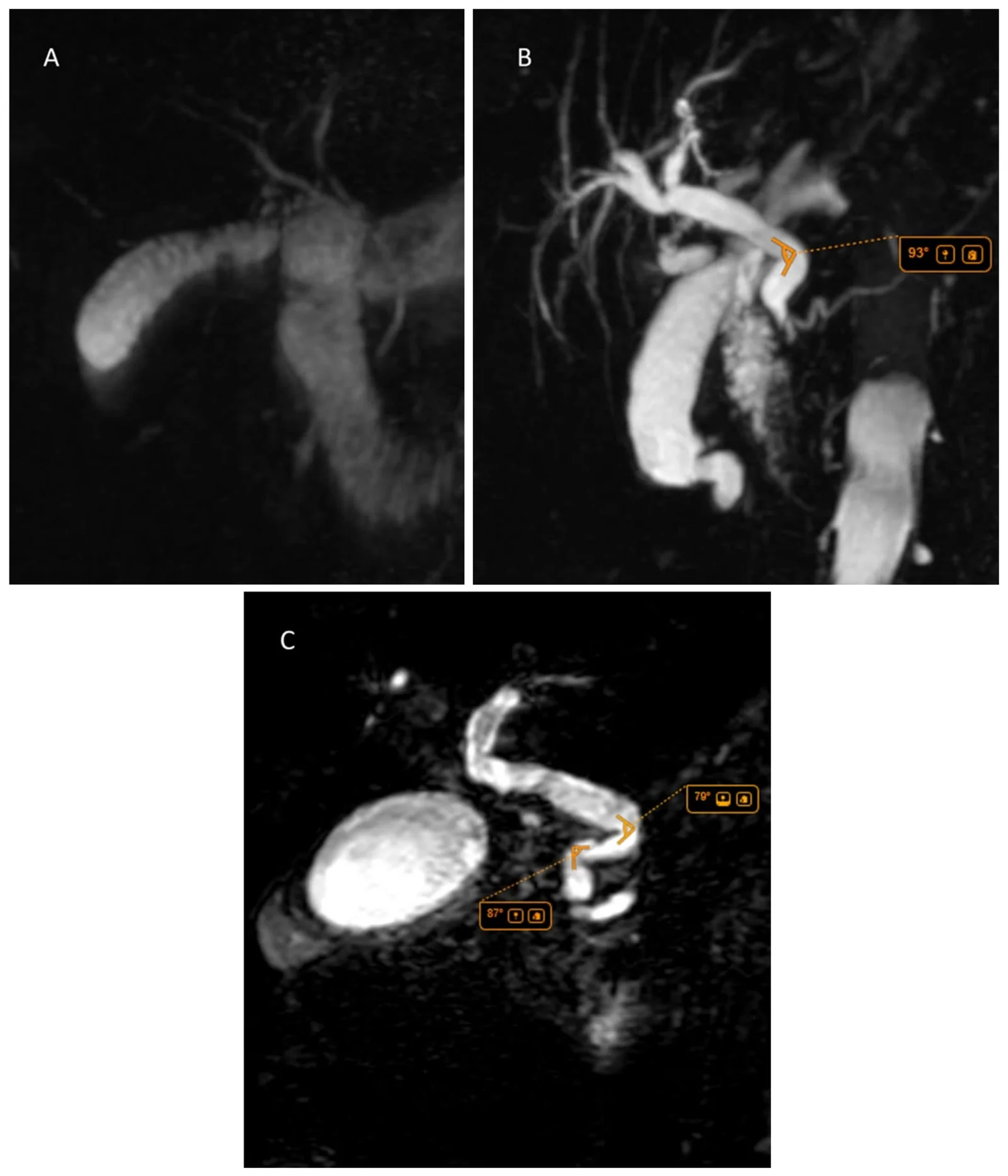
MRCP images illustrating CBD tortuosity grades: (**A**) grade 0, (**B**) grade 1, and (**C**) grade 2.

The three CBD tortuosity categories are illustrated in Figure 4.

#### 2.4.3. Stone-Related Variables

Stone-related variables included the presence, number, location and size (minimum and maximum diameter, in millimeters) of stones in the common bile duct, gallbladder and cystic duct, as well as the microlithiasis pattern.

CBD stone presence was considered positive when CBD stone number was greater than zero. The number of CBD stones was categorized as 1–3 or multiple (>3).

Gallbladder and cystic duct stone presence was defined similarly.

**Figure 5:**
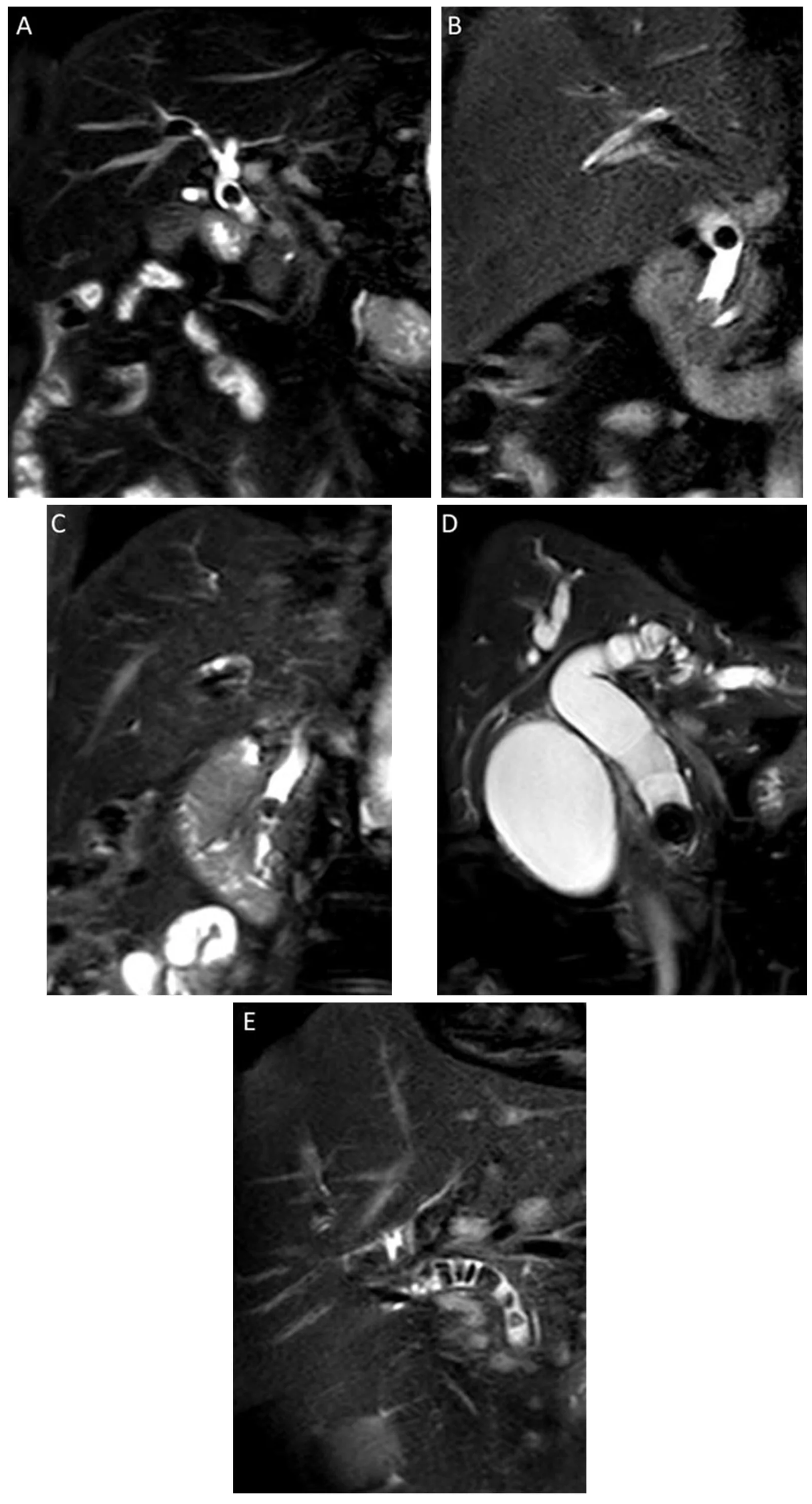
Coronal MRCP images illustrating CBD stone locations: (**A**) proximal, (**B**) middle, (**C**) distal, (**D**) impacted at the ampulla, and (**E**) multisegment.

Stone location was categorized using a six-point scale, reflecting the anatomical distribution along the common bile duct (0—absent; 1—proximal; 2—mid; 3—distal; 4—impacted ampullary; and 5—multisegment), as illustrated in Figure 5.

Proximal, mid and distal locations referred to upper, middle and lower third of the common bile duct.

Microlithiasis pattern was graded as absent (0) and present (1); microlithiasis was defined as more than three gallbladder stones, each measuring <3 mm in maximum diameter on MRCP images.

#### 2.4.4. Cystic Duct Parameters

Cystic duct parameters included insertion relative to the hepatic hilum (mm), cystic duct diameter (mm), and the presence and size (minimum and maximum diameter, in millimeters) of cystic duct stones.

Cystic duct diameter was measured at the best-visualized segment on MRCP reconstruction, avoiding ductal distortions caused by stones or artifacts.

**Figure 6:**
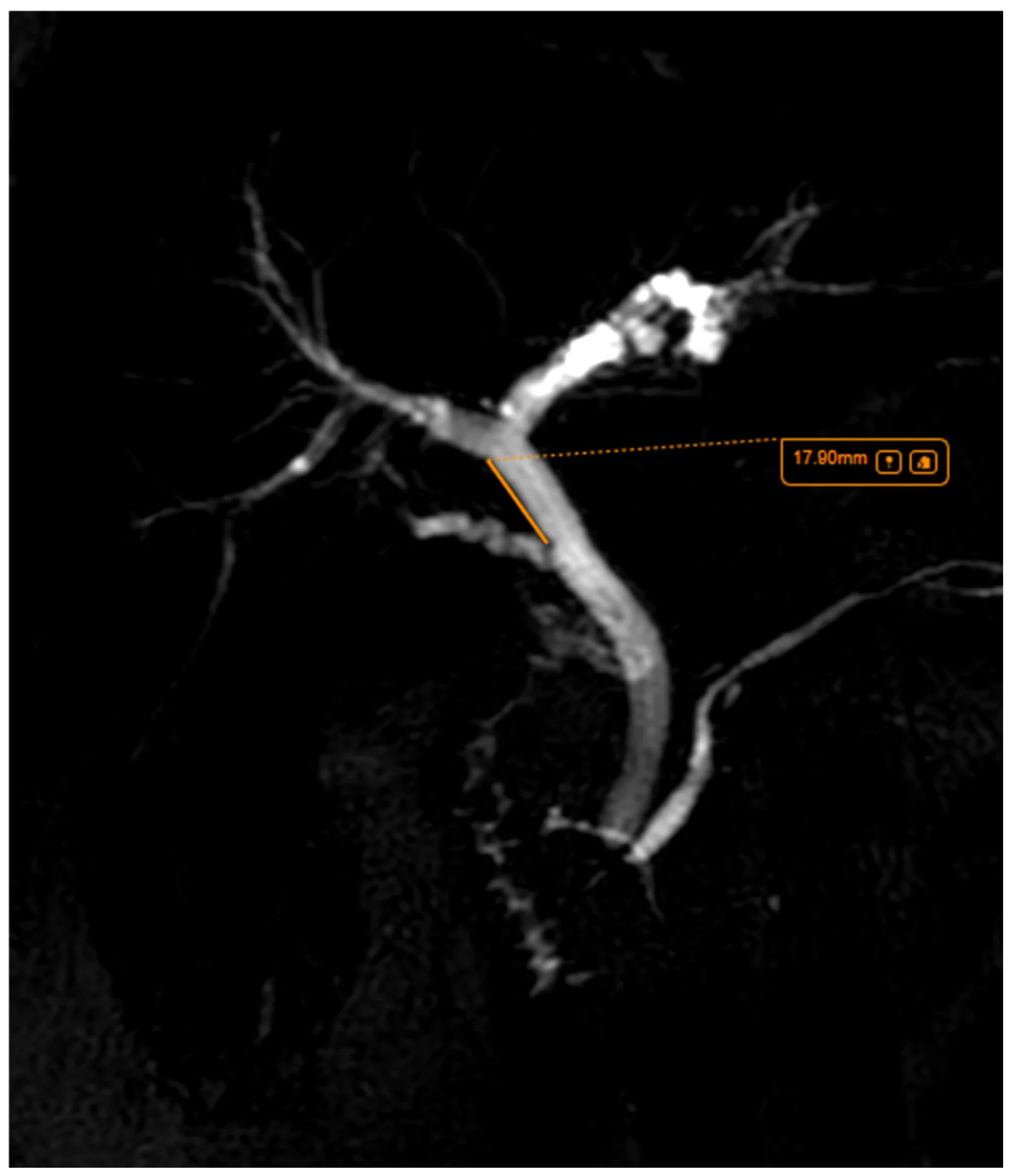
MRCP—cystic duct insertion distance from the hepatic hilum.

Cystic duct insertion was defined as the distance in millimeters from the hepatic hilum to the junction of the cystic duct and the common hepatic duct, using MRCP reconstructions, as shown in Figure 6.

#### 2.4.5. Gallbladder-Related Parameters

Gallbladder-related parameters included gallbladder status (present or prior cholecystectomy); in patients with gallbladder present, gallbladder-related variables included maximum gallbladder width (mm), wall thickness (mm) and the presence of pericholecystic fluid (0 = no, 1 = yes).

Within the study cohort, the gallbladder was present in 239 patients (98.8%), while 3 patients (1.2%) had a history of cholecystectomy.

#### 2.4.6. Associated Findings/Complications

Associated findings/complications included presence/absence of periampullary diverticulum and MRI signs of acute pancreatitis.

#### 2.4.7. Image Quality

Image quality was graded using a three-point scale (0—poor, 1—good, and 2—excellent) and was considered in the evaluation of imaging findings.

Excellent (2) image quality was defined as the presence of the following: complete visualization of extrahepatic duct from hilum to ampulla, distal CBD/ampulla well seen, minimal motion/ghosting, no relevant artifact and confident assessment of CBD stones, CBD diameter and cystic duct insertion.

Good (1) image quality was considered based on the presence of the following: adequate visualization of extrahepatic duct overall, but minor limitations, mild motion/overlap, distal CBD slightly degraded but still assessable, cystic duct insertion seen but not perfect and confident assessment of CBD stones yes/no and CBD diameter.

Poor (0) image quality defined as non-diagnostic distal CBD/ampulla, severe motion/artifact, cannot confidently exclude small stones or classify location and CBD diameter/level unreliable.

#### 2.4.8. Data Preparation

For analytical consistency, binary-derived variables were recalculated from the primary imaging fields rather than taken directly from spreadsheet-derived columns. CBD stone-size variables were analyzed only among CBD-positive patients and were not entered as predictors. For regression, GB maximum stone size was assigned a value of 0 when no GB stone was identified, allowing retention of stone-negative patients; the corresponding coefficient therefore represents a composite of stone absence/presence and increasing stone size rather than a purely within-stone size effect. Same-day liver function tests were not consistently available across the retrospective cohort and were not included in the dataset.

### 2.5. Statistical Analysis

Continuous variables were summarized as mean ± SD or median (IQR), according to distributional shape. Categorical variables were summarized as n (%). Normality was assessed using Shapiro–Wilk tests and visual inspection. Two-group comparisons used Mann–Whitney U tests for continuous/ordinal variables and chi-square or Fisher exact tests for categorical variables, as appropriate. Ordered age-stratum trends were assessed using Cochran–Armitage tests for proportions and Jonckheere–Terpstra permutation tests for continuous or ordinal variables. Correlations were evaluated with Spearman rho.

Multivariable logistic regression was performed with CBD stone presence as the endpoint. To limit overfitting, Model 1 used the following four predictor parameters selected from the original analysis plan: age per 10 years, sex, multiple GB stones, and GB maximum stone size. Model 2 added CBD diameter and IHBD score, for a total of six predictor parameters. With 65 CBD-stone events, the events-per-variable ratio was 16.3 for Model 1 and 10.8 for Model 2. Additional anatomical variables were retained for descriptive and group-comparison analyses and were not entered simultaneously in the primary adjusted model. Model discrimination was assessed using the area under the ROC curve (AUC), calibration with the Hosmer–Lemeshow test, and collinearity with variance inflation factors. Internal validation of Model 2 used 2000 bootstrap resamples to estimate optimism-corrected discrimination. All *p*-values were two-sided and rounded to three decimals; values below 0.001 are reported as *p* < 0.001. Analyses were implemented in Python 3.11 using pandas, SciPy, statsmodels, scikit-learn, matplotlib, and Graphviz.

## 3. Results

### 3.1. Cohort Characteristics and Overall MRCP Findings

A total of 242 patients were included in the final analysis. The median age was 63 years (IQR 49–73; range 11–90), and 135 patients (55.8%) were female. After recalculation of the derived variables from primary imaging fields, gallbladder stones were present in 211 patients (87.2%), while CBD stones were present in 65 patients (26.9%). Among CBD-positive patients, 43 had solitary CBD stones and 22 had multiple CBD stones.

The median CBD diameter for the entire cohort was 7.0 mm (IQR 6.0–8.9). IHBD dilatation was absent in 160 patients, mild in 53, moderate in 21, and severe in eight. Additional ancillary MRCP findings included CBD tortuosity in 54 patients (22.3%), periampullary diverticulum in 27 (11.2%), microlithiasis pattern in 14 (5.8%), cystic duct stones in 46 (19.0%), and MRI signs of acute pancreatitis in 12 (5.0%). Baseline characteristics are summarized in Table 1.

### 3.2. Age-Stratified Phenotype Shift

Age-stratified analysis showed differences in the distribution of gallbladder and ductal findings. As summarized in Table 2, GB stone prevalence decreased from 100.0% in patients younger than 20 years to 73.7% in patients aged ≥80 years, with a significant ordered trend (*p* = 0.006). In contrast, CBD stone prevalence increased from 12.5% in the youngest group to 42.1% in the oldest group (*p* = 0.038).

**Figure 7:**
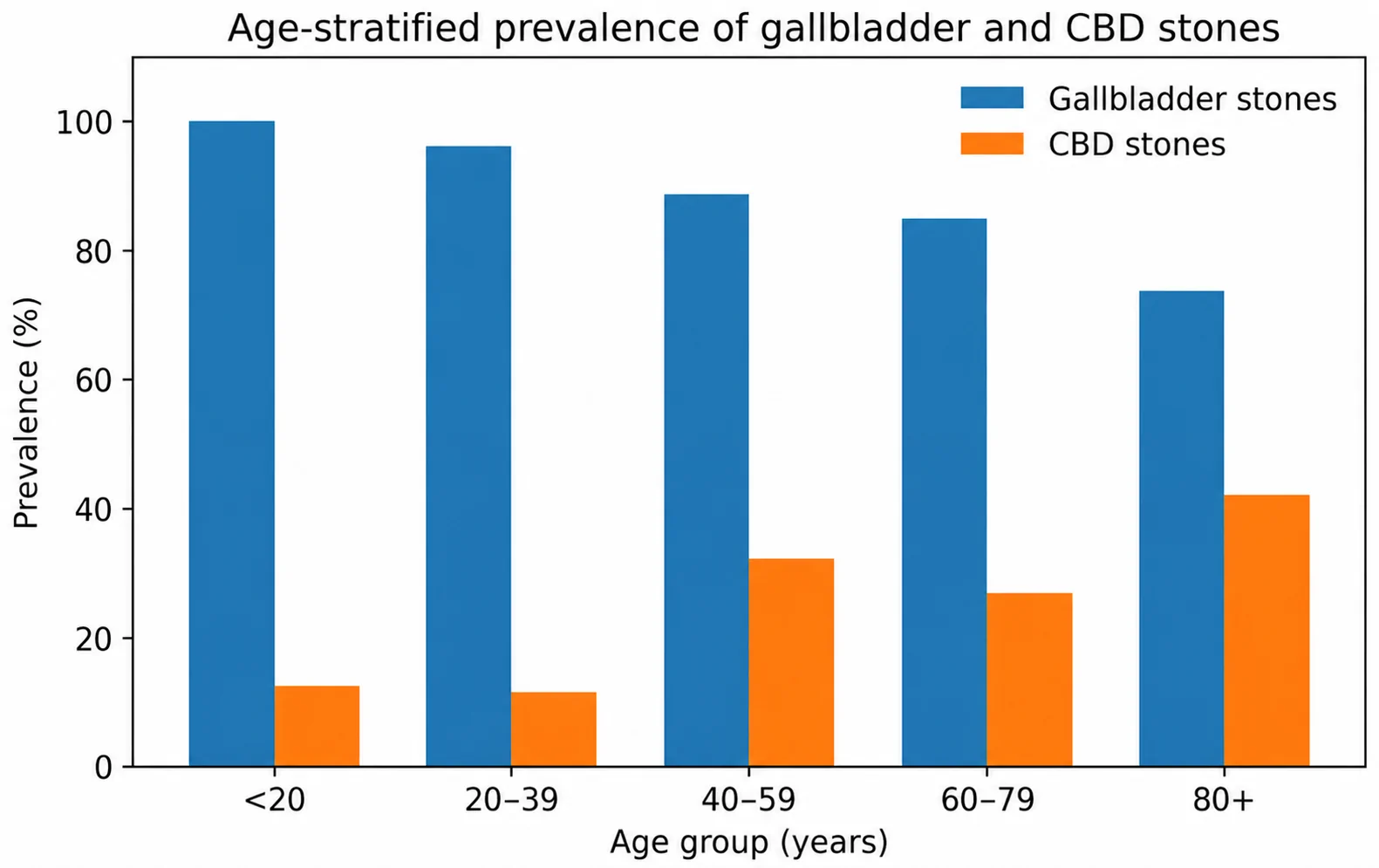
Age-stratified prevalence of gallbladder stones and CBD stones.

The contrasting age-associated prevalence patterns of gallbladder and ductal stones are illustrated in Figure 7. The oldest group had the lowest prevalence of GB stones and the highest prevalence of CBD stones.

**Figure 8:**
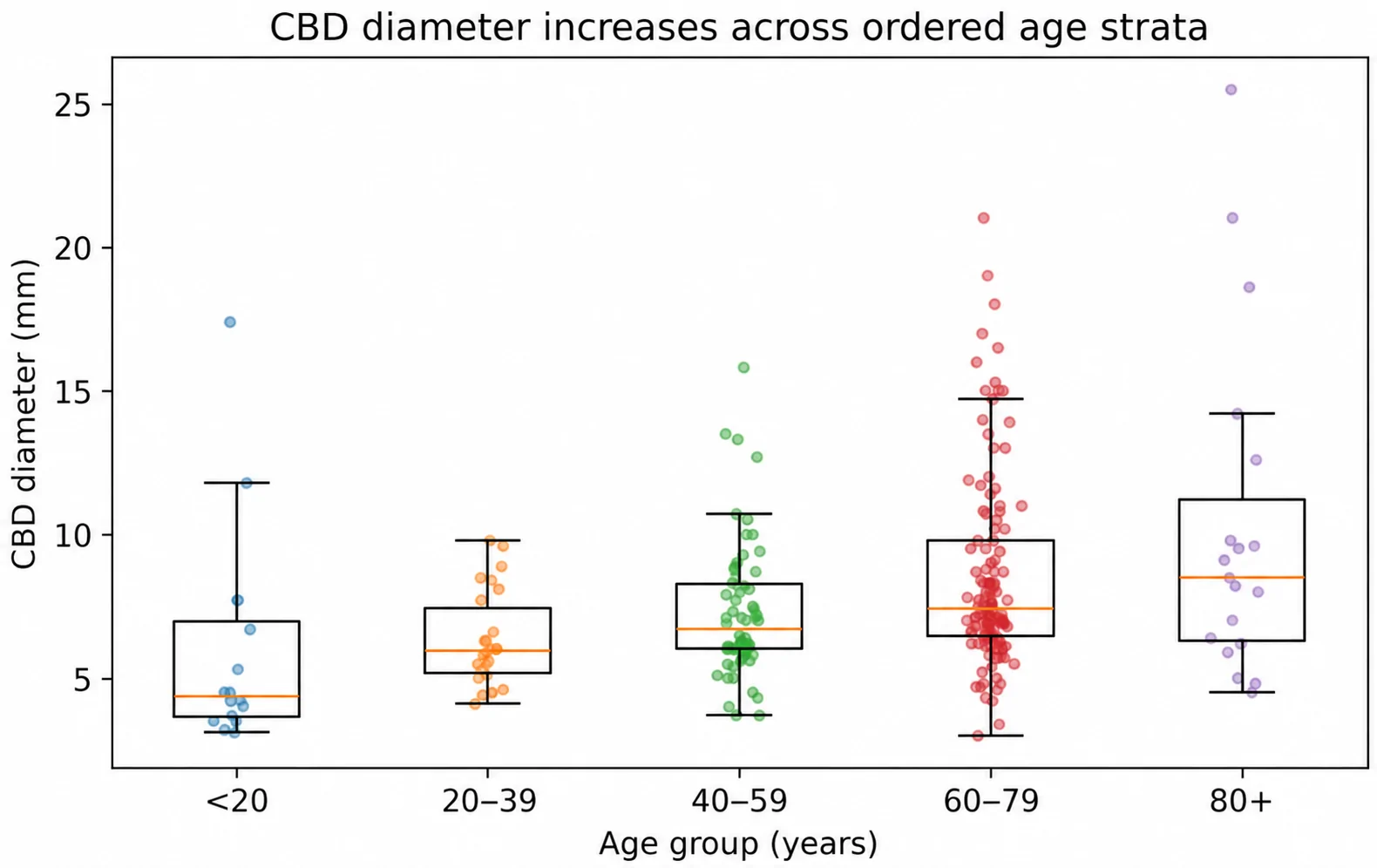
CBD diameter across ordered age groups.

CBD diameter showed the clearest age-associated gradient. It increased across ordered age strata (Jonckheere–Terpstra z = 5.45, *p* < 0.001), while IHBD dilatation also increased with age both as a binary finding (*p* = 0.004) and as an ordinal score (*p* < 0.001). The distribution of CBD diameter by age group is shown in Figure 8.

Additional age-associated differences were observed for CBD tortuosity score, cystic duct diameter, and gallbladder wall thickness. These differences were smaller than the variation in CBD diameter, identifying duct caliber as the main age-associated imaging finding.

### 3.3. CBD-Positive Versus CBD-Negative MRCP Findings

Patients with CBD stones differed from CBD-negative patients across several MRCP features. The main group comparisons are reported in Table 3. CBD-positive patients were older (median 66.0 vs. 62.0 years, *p* = 0.006) and showed substantially greater ductal involvement, with higher CBD diameter and IHBD scores.

**Figure 9:**
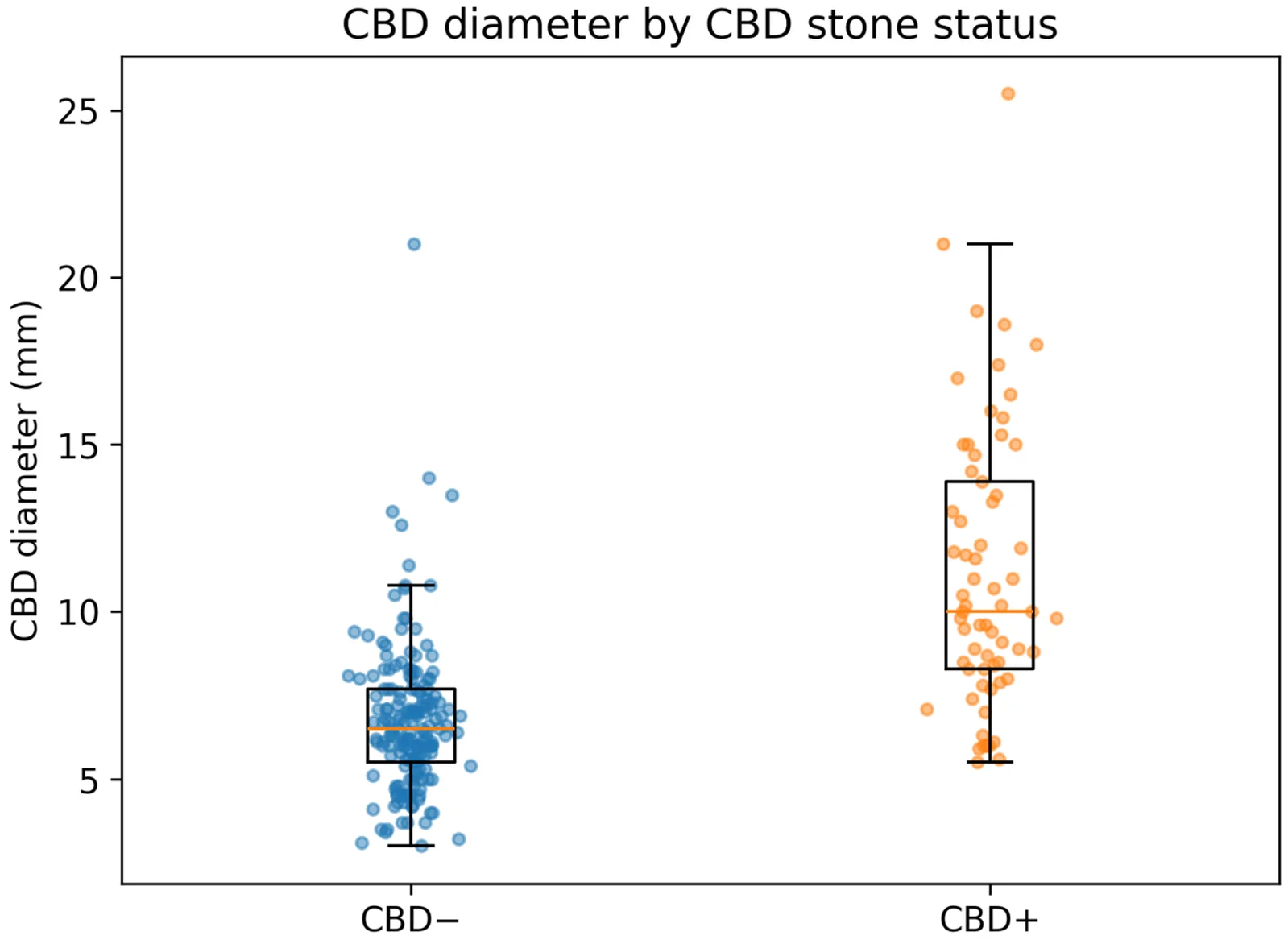
CBD diameter stratified by CBD stone status.

The most pronounced separation between groups was observed for CBD diameter. CBD-positive patients had a median CBD diameter of 10.0 mm (IQR 8.3–13.9) compared with 6.5 mm (IQR 5.5–7.7) in CBD-negative patients (*p* < 0.001), as shown in Figure 9.

**Figure 10:**
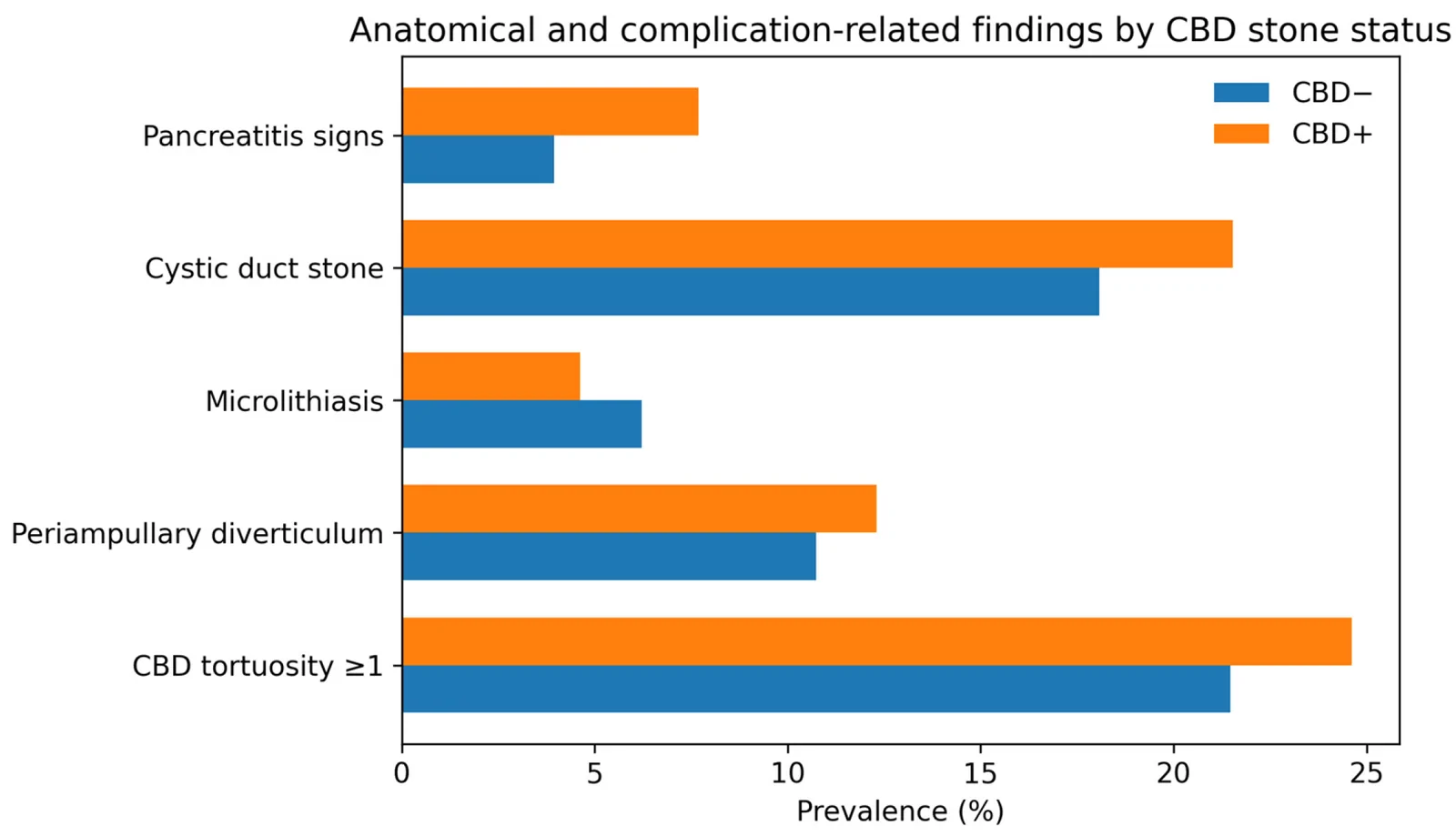
Additional anatomical findings according to CBD stone status.

Among the additional anatomical variables assessed, CBD-positive patients had a larger cystic duct diameter (median 6.4 vs. 5.0 mm, *p* < 0.001) and a greater cystic duct insertion distance from the hepatic hilum (median 20.9 vs. 16.0 mm, *p* = 0.010). Additional categorical anatomical findings according to CBD stone status are summarized in Figure 10.

These findings indicate that CBD stone status was primarily associated with greater ductal caliber and IHBD dilatation, while selected cystic duct measurements provided additional anatomical context.

### 3.4. CBD Stone Location and Ductal Associations

**Figure 11:**
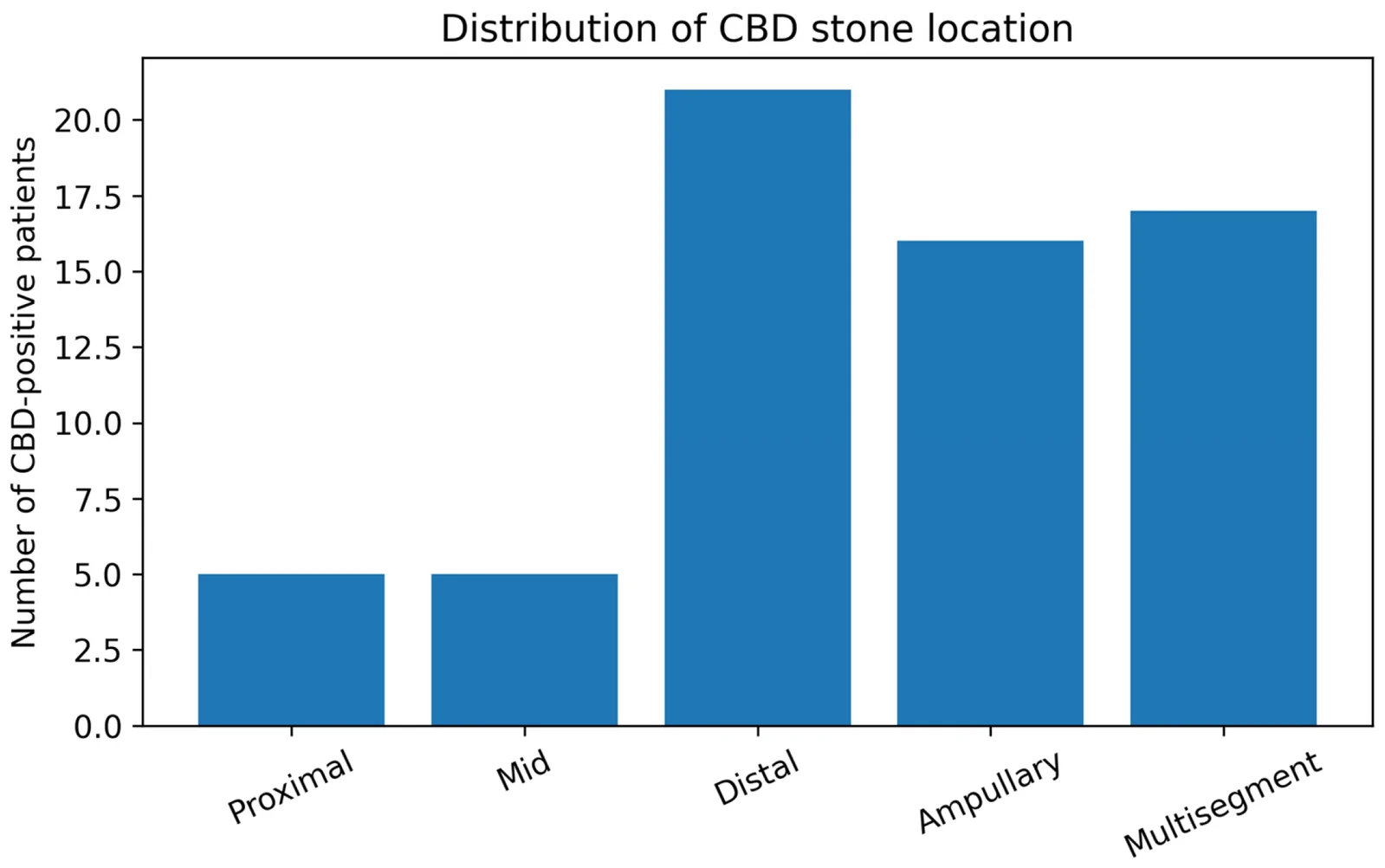
Distribution of CBD stone location among CBD-positive patients.

Among the 65 CBD-positive patients, distal or ampullary involvement predominated. Distal CBD stones were observed in 21 patients (32.3%), impacted ampullary stones in 16 (24.6%), and multisegment involvement in 17 (26.2%). Proximal and mid-CBD locations were less frequent. The distribution of stone location is shown in Figure 11.

Correlation analysis showed age-associated relationships among duct caliber and stone findings. Age correlated positively with CBD diameter (Spearman rho = 0.350, *p* < 0.001). CBD diameter also correlated with IHBD score and cystic duct diameter and, among CBD-positive patients, with maximum CBD stone size.

### 3.5. Multivariable Prediction of CBD Stones

To limit overfitting, the multivariable analysis used a parsimonious model structure. Model 1 included age, sex, multiple GB stones, and GB maximum stone size (four predictor parameters; 65 events; events-per-variable ratio 16.3) and showed moderate discrimination (AUC = 0.784).

Model 2 added CBD diameter and IHBD score (six predictor parameters; events-per-variable ratio 10.8). Discrimination improved to AUC = 0.896, with acceptable calibration (Hosmer–Lemeshow *p* = 0.213). The adjusted estimates from Model 2 are presented in Table 4.

**Figure 12:**
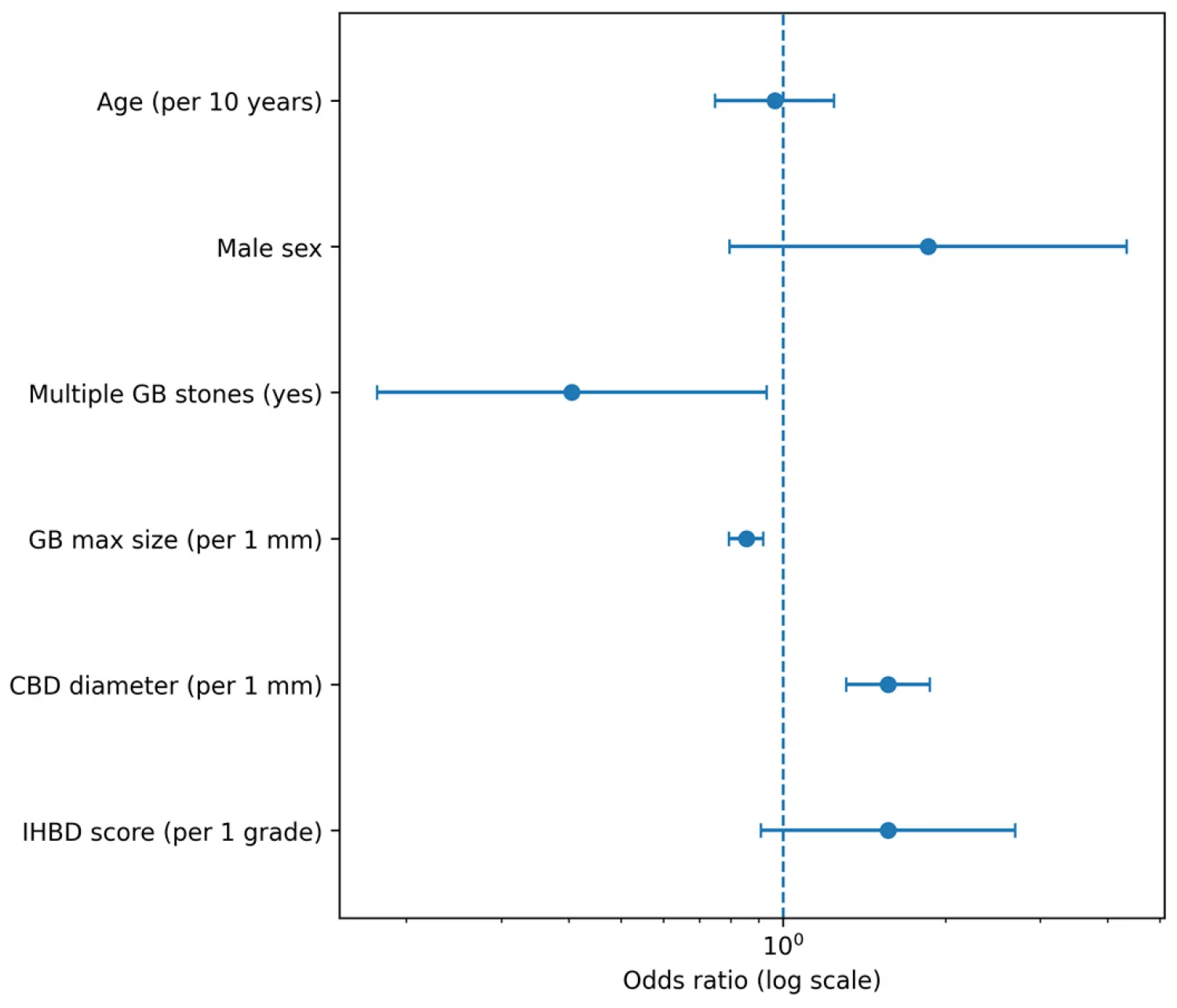
Forest plot of adjusted odds ratios from the parsimonious Model 2. The dashed line at OR = 1 indicates no association.

CBD diameter was the strongest positive imaging correlate of CBD stones (OR 1.567 per 1 mm, 95% CI 1.310–1.873, *p* < 0.001). Age was not independently associated after adjustment for duct caliber (OR 0.965 per 10 years, 95% CI 0.748–1.243, *p* = 0.781). The attenuation of the age coefficient indicates overlapping information between age and duct caliber but does not establish mediation. The adjusted odds ratios are displayed in Figure 12.

Multiple GB stones were associated with lower odds of CBD stones (OR 0.406, 95% CI 0.177–0.931, *p* = 0.033), and greater GB maximum stone size showed an inverse association (OR 0.854 per 1 mm, 95% CI 0.794–0.920, *p* < 0.001). These findings should be interpreted cautiously because GB maximum stone size was coded as 0 when no GB stone was present, and its coefficient is not a purely within-stone size effect.

**Figure 13:**
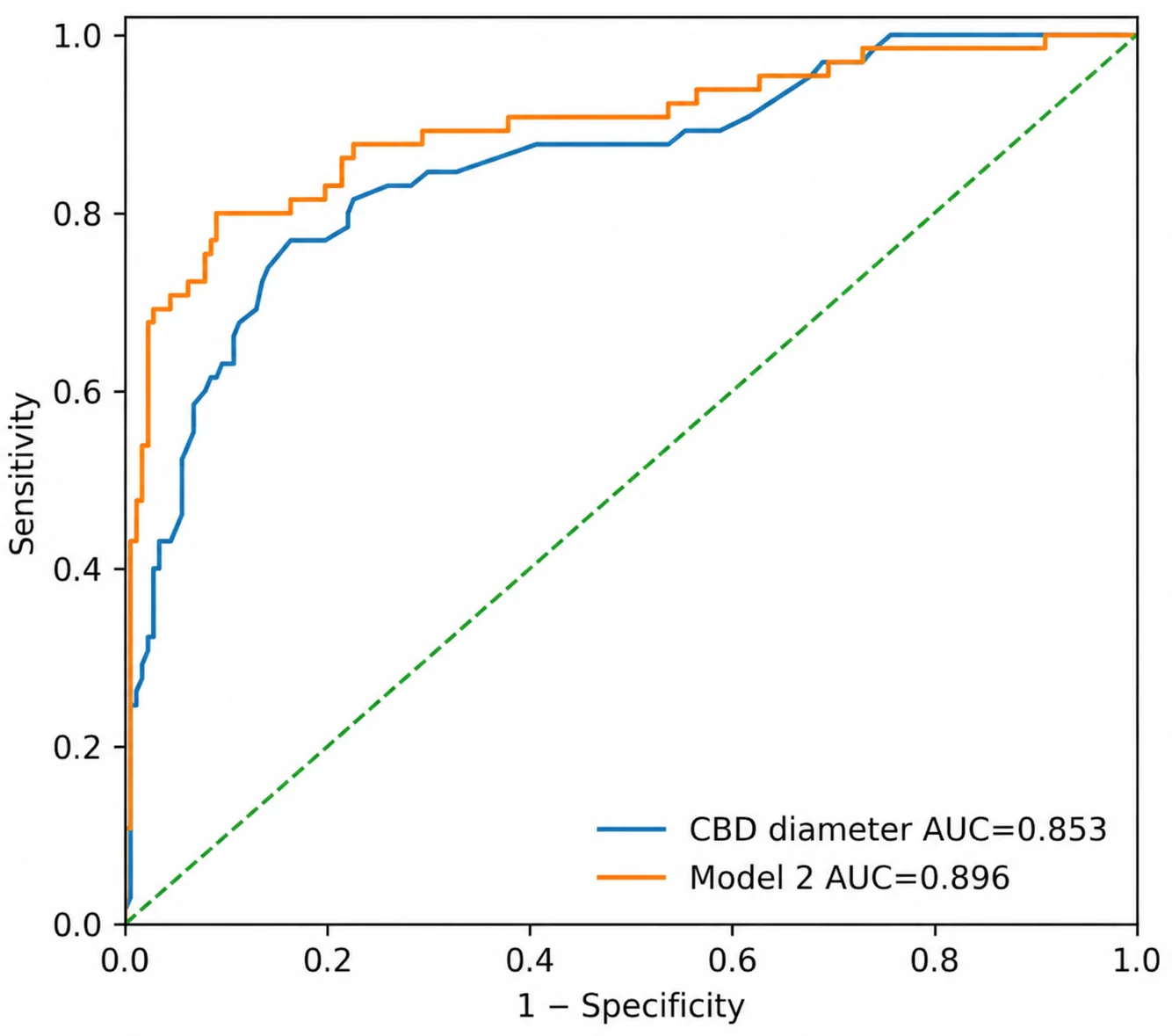
ROC curves for CBD diameter alone and the parsimonious Model 2.

ROC analysis was used to evaluate CBD diameter alone and the parsimonious Model 2. CBD diameter alone achieved an AUC of 0.853, while Model 2 achieved an AUC of 0.896. In 2000 bootstrap resamples, the optimism-corrected AUC was 0.884, indicating limited optimism in the apparent discrimination (Figure 13).

Clinically relevant CBD diameter thresholds are shown in Table 5. A 6.0 mm threshold was highly sensitive (95.4%) but poorly specific (32.2%), whereas a 10.0 mm threshold was highly specific (94.4%) but less sensitive (52.3%). The Youden-optimized threshold was approximately 8.3 mm, providing a balanced sensitivity of 76.9% and specificity of 83.6%.

Together, the regression and ROC analyses identified CBD diameter as the strongest imaging marker associated with CBD stones. The parsimonious model retained good discrimination after bootstrap correction, while gallbladder stone morphology provided additional but smaller information.

## 4. Discussion

In this consecutive MRCP cohort, older age groups had a lower prevalence of gallbladder stones and a higher prevalence of CBD stones, larger CBD caliber, and more frequent IHBD dilatation. This distribution is best described as an age-associated cross-sectional imaging pattern rather than a temporal change within the same patients. Gallstone disease becomes more common with age, but the balance between gallbladder and ductal findings is not uniform across age groups [11,12,13,14]. The present study adds an MRCP-based description from routine radiology practice, in which gallbladder morphology, duct caliber, and additional anatomical findings were evaluated together. This distinction is important because a referred MRCP cohort is enriched for symptomatic and diagnostically complex cases and should not be interpreted as a population prevalence sample.

The strongest result was the association between CBD diameter and CBD stones. CBD diameter increased across age strata, differed markedly between CBD-positive and CBD-negative patients, correlated with maximum CBD stone size among positive cases, and remained the strongest imaging correlate in the adjusted model. The age coefficient was no longer significant after CBD diameter was included. Because the study was cross-sectional, this attenuation should be interpreted as overlap in the statistical information carried by age and duct caliber. It does not demonstrate that CBD dilatation mediates an effect of age or establishes the temporal order of these findings. The finding nevertheless narrows the interpretation as follows: within this dataset, duct caliber carried more adjusted information about CBD stone status than chronological age alone.

These findings should be considered alongside published age-dependent reference limits for CBD diameter. Ultrasound and MRCP studies in asymptomatic populations have shown gradual widening of the CBD with age [8,9,10,15,16]. Such reference values are useful for avoiding overdiagnosis of obstruction in older individuals. However, a reference-range interpretation and a disease-oriented interpretation address different questions. In patients referred for biliary symptoms or suspected obstruction, a widened CBD remains relevant when it is accompanied by IHBD dilatation, a filling defect, or an abnormal distal duct. The present data therefore support contextual interpretation rather than reliance on a single universal diameter cutoff. The same measured diameter may therefore have different significance depending on symptoms, prior cholecystectomy, laboratory abnormalities, and associated ductal signs.

This point is clinically relevant because current guidelines recommend MRCP or EUS for patients with an intermediate likelihood of choledocholithiasis and reserve ERCP mainly for therapy [17,18,19,20,21,22,23]. MRCP is highly specific for ductal stones, although very small stones, sludge, and impacted ampullary calculi may remain difficult to identify [20,21,22]. In that setting, CBD caliber can serve as an indirect imaging clue that prompts careful review of the source images, distal CBD, ampullary region, and adjacent pancreaticoduodenal structures, where uncommon lesions may create diagnostic ambiguity because of their close relationship to the Vater papilla, pancreatic duct, and bile duct [24]. It should not, however, be used as a stand-alone diagnosis of choledocholithiasis, particularly in older or previously operated patients in whom duct caliber may be increased for other reasons. When MRCP remains equivocal despite persistent clinical suspicion, EUS is an important complementary test because it can identify small distal stones that may be inconspicuous on MRCP [17,18,19,20,21,22].

The ROC findings further illustrate this distinction. CBD diameter alone showed good discrimination for the consensus MRCP-defined outcome (AUC 0.853), while the parsimonious Model 2 achieved an apparent AUC of 0.896 and an optimism-corrected AUC of 0.884. These values describe separation within the present dataset and should not be interpreted as diagnostic accuracy against ERCP or surgery. The approximately 8.3 mm Youden threshold provided a balance between sensitivity and specificity, whereas 6.0 mm favored sensitivity and 10.0 mm favored specificity. All of these thresholds are exploratory and require external validation before clinical use. The high negative predictive values at lower thresholds may be useful descriptively, but predictive values are prevalence-dependent and cannot be transferred directly to populations with different referral patterns.

Gallbladder stone morphology provided additional, but smaller, information. Multiple GB stones and greater GB maximum stone size were inversely associated with CBD stones in the adjusted model. This finding is compatible with reports suggesting that smaller stones may pass more readily through the cystic duct [25,26,27,28]. Nevertheless, the present study did not document stone migration over time, previous spontaneous passage, or complete surgical history. In addition, GB maximum stone size was coded as 0 in stone-negative patients to retain the full cohort, so its coefficient combines information on stone absence or presence with increasing size. The association should therefore be considered hypothesis-generating rather than a direct estimate of migration risk. The inverse association may also reflect previous passage as follows: patients presenting with a CBD stone may have fewer or smaller residual GB stones at the time of MRCP. This possibility cannot be separated from stone morphology in a single cross-sectional examination.

The expanded dataset also allowed assessment of cystic duct anatomy and CBD stone location. Cystic duct diameter and insertion distance differed between CBD-positive and CBD-negative groups. These variables were not retained in the parsimonious primary model because the number of candidate predictors was large relative to the 65 CBD-stone events; their independent contribution should be assessed in a larger cohort. Distal, impacted ampullary, and multisegment stones accounted for most positive examinations. This distribution supports systematic inspection of the distal duct and periampullary region, particularly when the CBD is enlarged or upstream dilatation is present. The cystic duct is the anatomical route for secondary stone migration, so its caliber and insertion may be relevant even when they do not enter a stable multivariable model. Measurement reproducibility and anatomical variation should be evaluated before these parameters are proposed for routine risk assessment.

Periampullary diverticulum, CBD tortuosity, microlithiasis, cystic duct stones, pericholecystic fluid, and MRI signs of pancreatitis were not strong independent correlates in the primary analysis. Their absence from the final predictor set should not be read as evidence of no clinical relevance. Previous studies have linked periampullary diverticula, duct diameter, and duct angulation with biliary stasis or recurrent ductal stones [29,30,31,32,33,34,35]. In the present cohort, these variables were less frequent or showed smaller associations than CBD diameter. They are therefore better regarded as contextual MRCP findings that may become more informative in selected clinical subgroups or longitudinal studies. Their associations may also be more relevant to recurrence after duct clearance than to the presence of a stone at one MRCP examination. Longitudinal cohorts would be better suited to evaluate that question.

From a reporting perspective, the results support systematic documentation of CBD diameter, stone number and location, IHBD dilatation, and relevant cystic duct or periampullary findings. A structured description can improve communication among radiologists, gastroenterologists, and surgeons and can make the basis of an imaging impression more transparent. The clinical decision to proceed to EUS, ERCP, surgery, or follow-up should still integrate symptoms, laboratory findings, prior procedures, and pre-test probability. The present study did not evaluate management changes or patient outcomes and therefore cannot determine whether use of the proposed imaging pattern improves care. This approach avoids reducing interpretation to a binary stone-present or stone-absent statement and records the anatomical context that may explain equivocal findings or guide further evaluation.

Overall, the study identifies an age-associated pattern of greater ductal involvement and confirms CBD diameter as the strongest imaging correlate of CBD stones in this cohort. The results do not establish within-patient progression or a causal sequence. Future multicenter studies should combine standardized MRCP review with liver function tests, ERCP or surgical confirmation where appropriate, cholecystectomy history, and longitudinal outcomes. Such studies could determine whether age-specific reference limits, CBD caliber, and stone morphology improve prediction beyond established clinical pathways. Prospective follow-up would also help distinguish retained, recurrent, and spontaneously passed stones and would clarify whether the observed age-associated distribution has prognostic value.

The study has several strengths. It included a consecutive radiology-service cohort, applied predefined eligibility and imaging criteria, and evaluated a broad set of gallbladder, CBD, cystic duct, and periampullary variables. Ordered trend analyses, group comparisons, a parsimonious adjusted model, calibration assessment, and bootstrap internal validation provided complementary statistical checks. Review by two experienced radiologists with consensus resolution also ensured a consistent imaging classification across the cohort.

Several limitations should be acknowledged. The retrospective, single-center, cross-sectional design limits external validity and does not permit assessment of temporal sequence. Referral patterns and local MRCP protocols may also influence the observed prevalence of stones and associated anatomical findings. Consequently, the age-associated differences cannot be interpreted as progression within individual patients or as evidence of causation. The very young and very old strata were relatively small, so estimates at the extremes of age are less precise than those for the middle groups.

CBD stone status was determined by consensus MRCP interpretation rather than systematic ERCP, EUS, surgical, or longitudinal confirmation. These data were available only for subsets of patients and could not be used as a uniform independent reference standard. The study therefore evaluated associations with MRCP-defined CBD stones and did not estimate sensitivity, specificity, or diagnostic accuracy against endoscopic or operative findings. Small stones or sludge may be missed on MRCP, while equivocal signal defects may be overcalled; without a uniform reference, the direction and magnitude of any misclassification cannot be quantified.

Same-day liver function tests, including bilirubin, alkaline phosphatase, gamma-glutamyl transferase, and transaminases, were not consistently available in the retrospective radiology dataset and were not analyzed. Clinical symptom severity, inflammatory markers, treatment decisions, and longitudinal outcomes were also unavailable for the full cohort. These omissions limit comparison with established clinical prediction algorithms and prevent assessment of the incremental value of imaging over laboratory data. In particular, bilirubin and cholestatic enzymes could have added clinically important information to the imaging model and may alter the apparent contribution of CBD diameter.

The examinations were assessed by consensus, and formal interobserver agreement was not quantified. The reproducibility of ordinal variables such as IHBD dilatation, CBD tortuosity, image quality, and stone-location classification therefore remains uncertain. Future studies should include blinded independent readings and agreement statistics, particularly for variables intended for structured reporting or predictive modeling. Consensus reading can improve internal consistency but may overstate reproducibility compared with independent reporting in routine practice.

Some variables also require cautious interpretation. Several ancillary findings were infrequent, reducing power to detect smaller associations. Multiple comparisons were performed without formal adjustment, so *p*-values close to 0.05 should be regarded as exploratory. CBD stone-size variables were analyzed only in positive cases, while GB maximum stone size was coded as 0 in stone-negative patients; the latter coefficient is therefore a composite measure rather than a purely within-stone size effect. The subgroup analyses of stone size were also based on fewer observations than the full-cohort comparisons, which widens uncertainty around those estimates.

Finally, the original candidate set was too large for 65 CBD-stone events. The primary adjusted analysis was therefore reduced to six predictor parameters, yielding an events-per-variable ratio of 10.8. Bootstrap correction indicated limited optimism in model discrimination, but internal validation does not replace external validation. The model and the data-derived CBD diameter thresholds should be tested in larger independent cohorts with laboratory, endoscopic, surgical, and outcome data before they are used for clinical prediction. Penalized regression and prospective sample-size planning should be considered in subsequent model-development studies.

## 5. Conclusions

In this consecutive MRCP cohort, older age strata showed greater ductal involvement, including higher CBD stone prevalence, larger CBD diameter, and more frequent IHBD dilatation, while GB stone prevalence was lower in the oldest group. These are cross-sectional associations and do not demonstrate within-patient progression.

CBD diameter was the strongest independent imaging correlate of CBD stones in the parsimonious model, and the age coefficient attenuated after adjustment for duct caliber. Routine reporting of CBD caliber and associated ductal findings is supported, but the model and the 8.3 mm threshold require external validation against clinical, laboratory, and endoscopic or surgical outcomes.

## Figures and Tables

**Table 1:** Baseline characteristics and overall MRCP findings.

Variable	Summary	Notes
Age, years	63.0 (49.0–72.8)	range 11–90
Female sex	135 (55.8%)	
MRCP image quality good/excellent	223 (92.1%)	non-diagnostic studies excluded before analysis
IHBD dilatation score, 0/1/2/3	160/53/21/8	ordinal 0–3
CBD diameter, mm	7.0 (6.0–8.9)	
CBD stones present	65 (26.9%)	derived from primary CBD stone fields
CBD stones: solitary/multiple	43/22	among all patients
CBD stone maximum size, mm	8.0 (6.2–10.9)	CBD-positive with available size
CBD stone location: distal/ampullary/multisegment	21/16/17	CBD-positive location categories
Gallbladder stones present	211 (87.2%)	derived from primary GB stone fields
GB stones: solitary/multiple	71/140	among all patients
GB maximum stone size, mm	10.7 (6.0–15.9)	GB-positive with available size
CBD tortuosity score 0/1/2	188/49/5	ordinal 0–2
Periampullary diverticulum	27 (11.2%)	
Microlithiasis pattern	14 (5.8%)	
Cystic duct stone present	46 (19.0%)	
Cystic duct diameter, mm	5.2 (4.0–6.7)	
Cystic duct insertion from hepatic hilum, mm	17.1 (12.1–25.5)	
Gallbladder maximum width, mm	27.5 (21.5–33.4)	n = 225
Gallbladder wall thickness, mm	3.5 (3.0–4.8)	n = 225
Pericholecystic fluid	43 (19.1%)	n = 225 available
Acute pancreatitis signs on MRI	12 (5.0%)	

**Table 2:** Age-stratified MRCP findings across ordered age groups.

Age Group	n	GB Stones, n (%)	CBD Stones, n (%)	CBD Diameter, Median (IQR), mm	IHBD Any Dilatation, n (%)	CBD Tortuosity ≥1, n (%)	Cystic Duct Stone, n (%)
<20	16	16 (100.0%)	2 (12.5%)	4.3 (3.7–7.0)	4 (25.0%)	2 (12.5%)	2 (12.5%)
20–39	26	25 (96.2%)	3 (11.5%)	6.0 (5.1–7.4)	8 (30.8%)	5 (19.2%)	4 (15.4%)
40–59	62	55 (88.7%)	20 (32.3%)	6.7 (6.0–8.3)	12 (19.4%)	10 (16.1%)	13 (21.0%)
60–79	119	101 (84.9%)	32 (26.9%)	7.4 (6.5–9.8)	45 (37.8%)	31 (26.1%)	24 (20.2%)
80+	19	14 (73.7%)	8 (42.1%)	8.5 (6.3–11.2)	13 (68.4%)	6 (31.6%)	3 (15.8%)

**Table 3:** Selected comparisons between CBD-negative and CBD-positive patients.

Variable	CBD-Negative	CBD-Positive	Test	*p*-Value
Age, years	62.0 (45.0–71.0)	66.0 (56.0–75.0)	Mann–Whitney U	0.006
CBD diameter, mm	6.5 (5.5–7.7)	10.0 (8.3–13.9)	Mann–Whitney U	<0.001
IHBD score	0.0 (0.0–0.0)	1.0 (0.0–2.0)	Mann–Whitney U	<0.001
Cystic duct insertion distance, mm	16.0 (11.7–23.8)	20.9 (13.6–29.6)	Mann–Whitney U	0.010
Cystic duct diameter, mm	5.0 (3.7–6.4)	6.4 (4.9–8.1)	Mann–Whitney U	<0.001
GB maximum stone size, mm	10.7 (6.0–16.0)	8.9 (6.0–12.8)	Mann–Whitney U	0.238
Gallbladder maximum width, mm	26.8 (21.4–32.7)	30.9 (23.8–40.1)	Mann–Whitney U	0.031
Male sex	75 (42.4%)	32 (49.2%)	χ^2^	0.341
GB stones present	177 (100.0%)	34 (52.3%)	χ^2^	<0.001
Multiple GB stones	118 (66.7%)	22 (33.8%)	χ^2^	<0.001
CBD tortuosity ≥1	38 (21.5%)	16 (24.6%)	χ^2^	0.602
Periampullary diverticulum	19 (10.7%)	8 (12.3%)	χ^2^	0.730
Microlithiasis pattern	11 (6.2%)	3 (4.6%)	Fisher exact	0.765
Cystic duct stone	32 (18.1%)	14 (21.5%)	χ^2^	0.543
Acute pancreatitis signs on MRI	7 (4.0%)	5 (7.7%)	Fisher exact	0.314

**Table 4:** Multivariable logistic regression Model 2 for CBD stone presence.

Predictor	OR (95% CI)	*p*-Value	Interpretation
Age (per 10 years)	0.965 (0.748–1.243)	0.781	Not independently associated
Male sex	1.858 (0.795–4.338)	0.152	Not independently associated
Multiple GB stones (yes)	0.406 (0.177–0.931)	0.033	Inverse association
GB max size (per 1 mm)	0.854 (0.794–0.920)	<0.001	Composite inverse association; see note
CBD diameter (per 1 mm)	1.567 (1.310–1.873)	<0.001	Strongest positive imaging correlate
IHBD score (per 1 grade)	1.566 (0.910–2.696)	0.105	Not independently associated
Microlithiasis pattern	0.588 (0.059–5.896)	0.652	Not independently associated
Cystic duct stone	1.143 (0.416–3.142)	0.795	Not independently associated
Cystic duct diameter (per 1 mm)	1.096 (0.927–1.295)	0.282	Not independently associated
Cystic duct insertion distance (per 1 mm)	1.017 (0.980–1.056)	0.360	Not independently associated
Acute pancreatitis signs	2.099 (0.417–10.559)	0.368	Not independently associated
CBD diameter (per 1 mm)	1.633 (1.333–2.002)	<0.001	Dominant positive predictor
IHBD score (per 1 grade)	1.308 (0.721–2.375)	0.377	Not independently associated

Note: GB maximum stone size was coded as 0 in patients without GB stones; its coefficient is therefore a composite association. Model 2 included six predictor parameters for 65 events (events-per-variable ratio 10.8).

**Table 5:** Diagnostic performance of selected CBD diameter thresholds.

CBD Diameter Threshold (mm)	Sensitivity	Specificity	PPV	NPV
6.0	95.4%	32.2%	34.1%	95.0%
7.0	87.7%	59.3%	44.2%	92.9%
8.0	78.5%	78.0%	56.7%	90.8%
10.0	52.3%	94.4%	77.3%	84.3%
8.3	76.9%	83.6%	63.3%	90.8%

## Data Availability

The raw data supporting the conclusions of this article will be made available by the authors on request.
